# Water Mobility in the Interfacial Liquid Layer of Ice/Clay Nanocomposites

**DOI:** 10.1002/anie.202013125

**Published:** 2021-02-25

**Authors:** Hailong Li, Julian Mars, Wiebke Lohstroh, Michael Marek Koza, Hans‐Jürgen Butt, Markus Mezger

**Affiliations:** ^1^ Department of Physics at Interfaces Max Planck Institute for Polymer Research Ackermannweg 10 55128 Mainz Germany; ^2^ Heinz Maier-Leibnitz Zentrum (MLZ) Technische Universität München Lichtenbergstrasse 1 85748 Garching Germany; ^3^ Institut Laue-Langevin 71 Avenue des Martyrs, CS 20156 38042 Grenoble France; ^4^ Department of Physics, Dynamics of Condensed Systems University of Vienna Boltzmanngasse 5 1090 Wien Austria

**Keywords:** interfacial premelting, permafrost, quasi-elastic neutron scattering, water mobility

## Abstract

At solid/ice interfaces, a premelting layer is formed at temperatures below the melting point of bulk water. However, the structural and dynamic properties within the premelting layer have been a topic of intense debate. Herein, we determined the translational diffusion coefficient D_t_ of water in ice/clay nanocomposites serving as model systems for permafrost by quasi‐elastic neutron scattering. Below the bulk melting point, a rapid decrease of D_t_ is found for charged hydrophilic vermiculite, uncharged hydrophilic kaolin, and more hydrophobic talc, reaching plateau values below −4 °C. At this temperature, D_t_ in the premelting layer is reduced up to a factor of two compared to supercooled bulk water. Adjacent to charged vermiculite the lowest water mobility was observed, followed by kaolin and the more hydrophobic talc. Results are explained by the intermolecular water interactions with different clay surfaces and interfacial segregation of the low‐density liquid water (LDL) component.

## Graphical abstract

Using quasi‐elastic neutron scattering (QENS), the mobility of water molecules confined in the premelting layer of three different ice/clay nanocomposites is quantified. Layers of low‐density liquid water are formed adjacent to ice Ih and clay mineral interfaces. This leads to a reduction of the translational diffusion coefficient within the premelting layer.

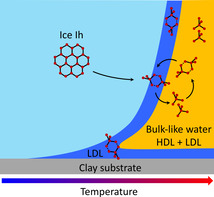

## Introduction

Interactions of water molecules and mineral surfaces play a major role in environmental sciences. At the involved water/solid interfaces, the hydrogen bonding network between neighboring water molecules is disturbed. Interface‐induced phase transitions are consequences of such modifications of the intermolecular force balance.^[1]^ Depending on the specific system, interface‐induced order and disorder has been observed.[^2^, ^3^] For ice in particular, interface‐induced premelting is found by experiments and simulations.^[4]^ In this case, an amorphous liquid layer near interfaces emerges below the bulk crystal/liquid phase transition. The presence of this nanoscopic liquid layer and its properties have important implications for friction^[5]^ as well as for macroscopic geophysical processes, as reviewed by Dash et al.^[6]^ Therefore, a molecular‐level understanding of the structure and dynamics within the liquid layer is highly desirable.

In 1859, Faraday proposed the existence of a liquid‐like layer at free ice surfaces.^[7]^ Since then, the growth law of this liquid layer, that is, its thickness *d*(*T*−*T*_m_) vs. temperature *T* below the bulk melting point *T*_m_, was extensively studied using various experimental techniques[^8^, ^9^, ^10^, ^11^, ^12^] and molecular dynamics simulations.[^13^, ^14^] However, unlike for the free ice surface[^15^, ^16^] only relatively little is known about thermodynamic, structural, and dynamic properties of this premelting layer at ice/solid interfaces. This includes materials parameters such as density, latent heat of melting, or viscosity and thermodynamic response functions such as compressibility, heat capacity, or dielectric constants.^[17]^ Moreover, properties on the molecular level, such as pair correlation functions, coordination number, rotational motion, energy transfer rates between adjacent water molecules connected by hydrogen bonds and their ability to form complexes and hydration shells, might deviate from bulk. However, due to a lack of experimental data, theoretical models[^18^, ^19^] rely on bulk values for the latent heat of melting or ion solubilities.

By X‐ray reflectivity, Engemann et al. found a quasiliquid layer with a 20 % higher density compared to bulk water.^[10]^ Recently, high‐density liquid water was observed at the interface between water and high‐pressure ice III or VI by optical microscopy.^[20]^ This indicates that the interfacial premelting layer and supercooled bulk water exhibit remarkable structural differences. Furthermore, such density increase also suggests changes in the water mobility within the premelting layer. For the viscosity, a strong increase relative to supercooled bulk water was found.^[21]^ While friction force measurements at ice/quartz interfaces gave an increase by more than one order of magnitude,^[22]^ quasi‐elastic neutron scattering (QENS)^[23]^ on graphitized carbon black indicated an increase by less than a factor of two. These observations are particularly important for the viscoelastic properties of partially frozen ice/solid composites, serving as model systems for permafrost.

Intense attention has also been paid to experimental observations of water diffusion in swollen clays.^[24]^ Depending on the water/solid interactions, molecular dynamics simulations of interfacial water adjacent to hydrophobic and hydrophilic surfaces indicate a relative increase or decrease of the diffusion constant, respectively.^[25]^ However, particle‐tracking studies in electric field gradients,^[26]^ nuclear magnetic resonance (NMR),^[27]^ and QENS^[24]^ provide no clear evidence that these results are transferable to the water mobility within the interfacial premelting layer. Therefore, despite its importance, the understanding of the surface and interfacial melting of ice, in particular the molecular scale dynamics within the premelting layer, is still under debate.

Hydrogen exhibits a large incoherent neutron scattering cross section.^[28]^ This makes QENS an ideal technique to provide information on the dynamics of liquid water.[^29^, ^30^] In addition to bulk measurements, QENS is also an ideal tool to quantitatively study water dynamics in wet clay minerals,[^31^, ^32^, ^33^, ^34^] surface melting of adsorbed multilayer films,[^35^, ^36^, ^37^] or interfacial ice melting in powders with high surface to volume ratio.^[23]^

Herein, we studied the translational water diffusion coefficient *D*_t_ in the interfacial premelting layer of ice/clay nano‐composites. The clay platelets have a planar geometry, large surface‐to‐volume ratios, and a molecular scale surface roughness. This ensures that the formation of the premelting layer is governed by intrinsic interfacial premelting rather than the Gibbs–Thomson effect. The Gibbs–Thomson effect is a change in the melting point due to curvature of the interface. Therefore, it strongly affects premelting in ice nano‐crystals,^[38]^ spherical nano‐powders,^[39]^ or nano‐pores.^[40]^

### Experiments

Temperature dependent QENS experiments (stability ±0.01 °C) were carried out from −100 °C. Subsequently, the temperature was gradually increased to above the bulk melting point. To elucidate the influence of the water/solid interactions, we compare QENS results from the charged hydrophilic clay vermiculite, the uncharged hydrophilic clay kaolin, and the more hydrophobic clay talc. Detailed sample preparation can be found in the SI. The preparation of the clay minerals and their morphology characterization by scanning electron microscopy and atomic force microscopy has been reported previously.^[41]^ Quantitative analysis of nitrogen adsorption isotherms using a Brunauer‐Emmett‐Teller (BET) slit model gave a specific surface area of 10.5 m^2^ g^−1^ (vermiculite), 10.2 m^2^ g^−1^ (kaolin), and 4.9 m^2^ g^−1^ (talc) (Figure S1). The water content was determined by thermogravimetric analysis (TGA/DSC 3+, Mettler Toledo) to be 33.6 wt % (vermiculite), 17.6 wt % (kaolin), 17.4 wt % (talc I), and 16.2 wt % (talc II), respectively. For the QENS measurements, wet clay samples of approx. 0.5 mm thickness were contained in flat, rectangular aluminum cells.

## Results and Discussion

**Figure 1:**
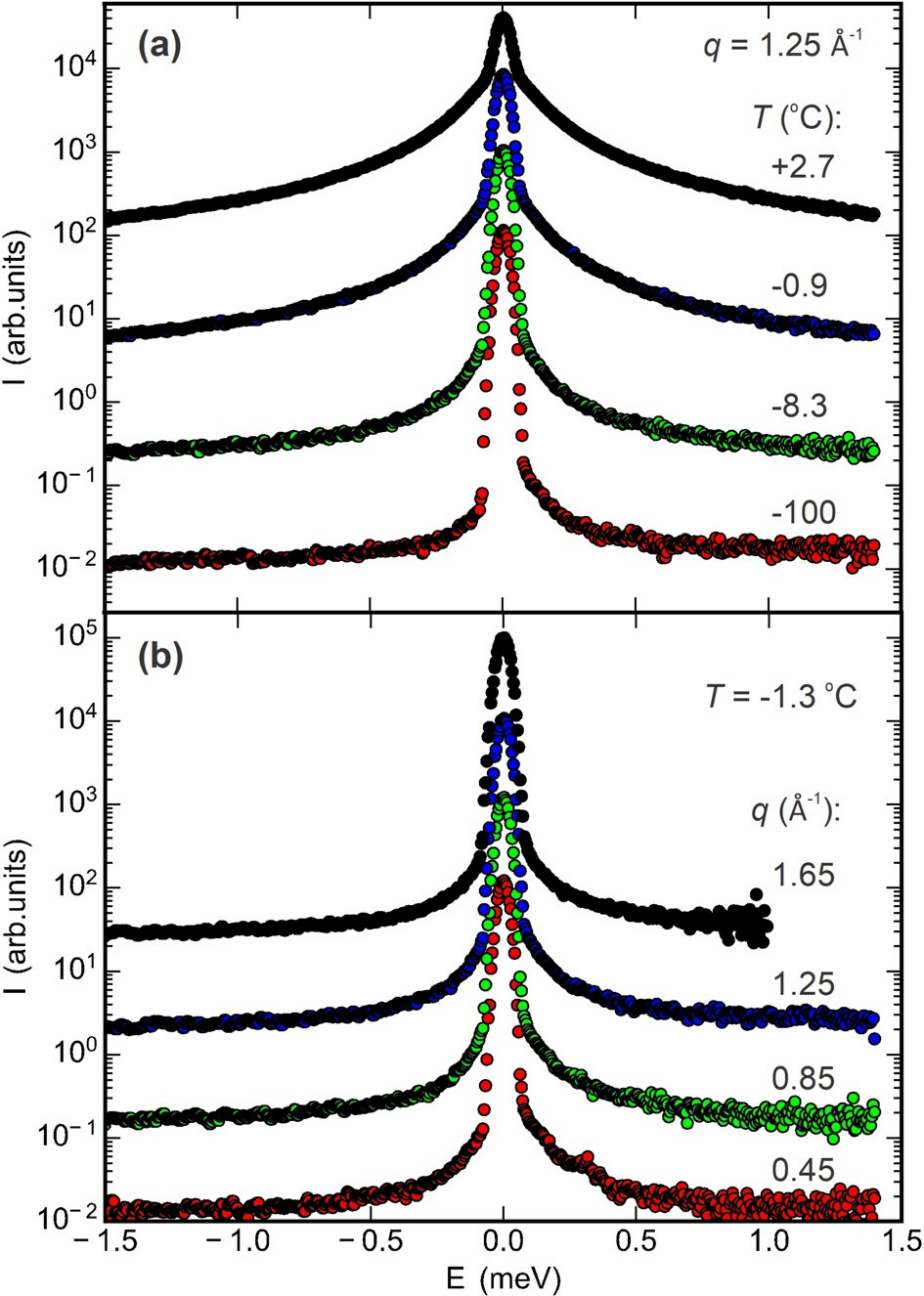
QENS spectra of the kaolin/water composite sample. a) Spectra at a momentum transfer *q*=1.25 Å^−1^ above and below the bulk melting point; b) *q* dependency at a temperature of −1.3 °C. All curves are vertically shifted by a factor of 10 for clarity.

Representative QENS spectra from the kaolin/water composite sample at *q*=1.25 Å^−1^ in the temperature range between −100 °C and +2.7 °C are shown in Figure 1 a. With increasing temperature, the intensity in the broad wings (quasi‐elastic peak) around the elastic peak at *E*=0 meV increases. This indicates the gradual growth of the liquid layer as temperature approaches the bulk melting point of water. Figure 1 b shows the spectra at −1.3 °C for four different momentum transfers *q* between 0.45 Å^−1^ and 1.65 Å^−1^. As expected for translational diffusive motion, with increasing *q* the wings broaden [Eq. (1) and Ref. [42]]. Similar behavior was observed in QENS spectra of talc/water and vermiculite/water samples (Figure S2 and S3).

To quantitatively evaluate the dynamics of water molecules and extract the translational diffusion coefficient *D*_t_ of the interfacial liquid layer, a model‐free approach was used to consistently analyze all the QENS spectra with momentum transfers *q*=0.45 Å^−1^ to 1.65 Å^−1^ at a given temperature.^[42]^ In this approach, the quasi‐elastic signal is composed of two Lorentzian functions *L*_r_(*q*,*ω*) and *L*_t_(*q*,*ω*). They represent fast rotational and slow translational jump diffusion of water molecules. The *q*‐dependent linewidth *Γ*_t_(*q*) of the narrow (slow) Lorentzian spectral component *L*_t_(*q*,*ω*) was constrained by Equation 1, assuming a constant apparent jump length *l*=0.77 Å according to Qvist et al.^[42]^ (Figure S6). Details of the fit functions and analysis procedure are summarized in the SI.

(1) $$\Gamma_{t}\left(q\right)=\frac{D_{t\;}q^{2}}{1+{\left(ql\right)}^{2}/6}$$

**Figure 2:**
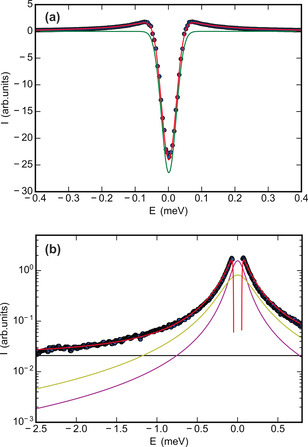
Measured (blue circles) and calculated (red curve, [Eq. S9]) QENS difference spectrum for the ice/kaolin composite sample at −1.3 °C and *q*=1.25 Å^−1^. a) Elastic contribution (green curve, [Eq. S3]); b) quasi‐elastic components for the translational diffusive motion *L*_t_(*q*,*ω*) (purple curve, [Eq. S5]), fast component *L*_r_(*q*,*ω*) (yellow curve, [Eq. S6]), and constant term *C* (black line) highlighted by logarithmic scaling. All calculated curves are convoluted with the Gaussian instrumental resolution function given in Equation S2.

To emphasize the QENS signal from the interfacial premelting layer, difference spectra were calculated by subtraction of the signals recorded on the completely frozen samples at −100 °C. Fitting of these difference spectra results in a stable analysis procedure. For all datasets, the measured patterns are perfectly reproduced. Figure 2 shows the experimental data (blue points) and the calculated spectra *I*(*E*) (red curve) for the ice/kaolin composite sample at −1.3 °C and 1.25 Å^−1^ momentum transfer. The green curve in Figure 2 a shows the elastic component. Its Gaussian line shape is given by the instrumental resolution. Figure 2 b summarizes the contributions to the quasi‐elastic signal. For each temperature, the FWHM *Γ*_r_ of the broad (fast) Lorentzian component (yellow curve) was constrained to a common value within a *q* series. Using this procedure, robust parameters that exhibit a consistent temperature variation were obtained for all ice/clay composite samples.

**Figure 3:**
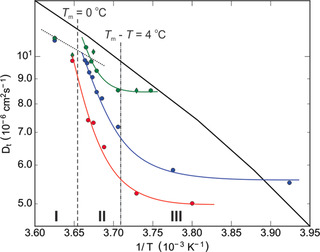
Translational diffusion coefficient *D*_t_ of water extracted from the slow component of the QENS spectra of talc I (green diamonds), talc II (green circles), kaolin (blue circles), and vermiculite (red circles) composite samples. Literature values of liquid bulk water are shown for comparison (black curve).^[42]^ Vertical lines indicate −4 °C (dash‐dotted) and the bulk melting point at 0 °C (dashed). Lines are guides to the eye.

Figure 3 summarizes the translational diffusion coefficient *D*_t_ of the premelting water fraction extracted from QENS measurements on the talc, kaolin, and vermiculite samples (symbols) in an Arrhenius plot. Values of supercooled liquid bulk water, determined by Qvist et al.^[42]^ (black curve), are given for comparison.

Below the bulk melting point of water, a significant slowdown of the translational diffusion is observed in all clay composites. This effect is most pronounced for the charged hydrophilic vermiculite. In contrast, for the uncharged talc a smaller slowdown of less than 11 % is found. While the observed reduction of *D*_t_ is highly significant, its values clearly show that for all cases studied here the interfacial premelting layer is liquid. These values are similar to the results reported by Maruyama et al. for hydrophobic graphitized carbon black.^[23]^

At low temperatures, that is, *T*_m_−*T*>4 K (Figure 3, region III), the values of *D*_t_ extracted from the QENS data indicate a flattening of the curves. In this temperature region, an effective interfacial premelting layer thickness smaller than 1.4 nm and 2.0 nm was determined by high‐energy X‐ray diffraction^[41]^ for ice/vermiculite and ice/kaolin samples, respectively. These values approach characteristic structural water dimensions. A thickness of 2.0 nm corresponds to approx. seven 2.8 Å monolayers of liquid water^[43]^ or three times the 0.736 nm lattice constant of ice Ih along its *c* axis, that is, perpendicular to the basal plane.^[44]^ On the other hand, this thickness is significantly larger compared to swollen clays where only one or two water monolayers are intercalated in between clays.[^31^, ^32^, ^33^, ^34^]

For 0<*T*_m_−*T*<4 K (Figure 3, region II), a strong increase of *D*_t_ with increasing temperature *T* is observed. Experimental studies showed that, at these temperatures, the premelting layer is rapidly thickening.[^6^, ^9^, ^10^, ^11^, ^41^] Therefore, with increasing temperature the geometric confinement effect on the water molecules inside the premelting layer will decrease.

Above its bulk melting point *T*_m_ (Figure 3, region I), the ice confined between clay platelets is totally molten. From the water fraction in the clay composites and specific surface area we estimate an average water layer thickness of 97 nm (vermiculite), 42 nm (kaolin), 86 nm (talc I), and 79 nm (talc II) between the clay platelets. A relatively small decrease of the translational water diffusion coefficients *D*_t_ by approx. 12 % is observed for talc and kaolin samples (Figure 3, region I). This arises from the contribution of the small fraction of less mobile interfacial water.

It is expected that the water mobility within the interfacial premelting layer is affected by the interactions between premelting water molecule and the clay surfaces. Bare kaolin surfaces have a surface energy of 171 mN m^−1^ with a ratio of 40 % dispersive and 60 % nondispersive interactions.^[45]^ The positive spreading coefficient of 76 mN m^−1^, calculated by the Fowkes method,^[46]^ reflects the strong hydrophilic nature of kaolin surfaces. In contrast, the attraction of water molecules to the more hydrophobic talc is significantly smaller. At low temperature, that is, thin premelting layers (Figure 3, region III), the mobility of water molecules in the premelting layer follows the trend: *D*_vermiculite_<*D*_kaolin_<*D*_talc_. Therefore, the more hydrophilic the clay mineral, the stronger the *D*_t_ decrease within the premelting layer.

The Netz group investigated the water dynamics near hydrophobic and hydrophilic membranes by molecular dynamics simulations.^[25]^ For distances between water molecules and the hydrophilic membrane smaller than 1 nm, the water diffusion coefficient *D*_t_ decreases dramatically. On the other hand, near hydrophobic surfaces a small increase of *D*_t_ was found. However, for larger wall separations *D*_t_ quickly approaches its bulk value. These findings are consistent with our observation that at *T*_m_−*T*<4 K *D*_t_ increases rapidly. Since none of our clays are strongly hydrophobic, the weak increase of the diffusion constant near hydrophobic interfaces, predicted by simulations,^[25]^ is not found.

The nano‐confinement effect on water dynamics in reverse micelles was investigated by the Fayer group using ultrafast infrared pump‐probe spectroscopy.[^47^, ^48^, ^49^] For small reverse micelles with diameters *d*≤2.5 nm, they observed spectral signatures from a single water ensemble only. These results suggest that in the temperature region III (Figure 3) there is only one liquid water ensemble present in the nanoscopic premelting layer of clay composites. This observation is consistent with the flattening of the *D*_t_ curve in region III. However, for larger spherical pores or slit pores with sizes 2.5 nm≤*d*≤5.5 nm, two water ensembles have been found.[^47^, ^48^, ^49^] The first one comprises the core, the second one includes the water molecules adjacent to the interface. Likewise, with increasing premelting layer thicknesses, contributions from the fast‐translational water diffusion apart from the clay surface will gradually start to dominate the average QENS signal. This readily explains the rapid increase of *D*_t_ observed in region II of Figure 3.

The properties and nature of the premelting layer formed at ice surfaces and interfaces is a topic of intense debate. Smit et al. found that the sum‐frequency generation spectra (SFG) from the ice premelting layer and supercooled bulk water are indistinguishable.^[50]^ Therefore, they deduced that the surface of ice is more like supercooled liquid water down to 245 K.^[50]^ However, using the same experimental technique Sánchez et al.^[12]^ found that the SFG response from ice surfaces at 270 K is different compared to supercooled water at the same temperature, but more similar to that of ice at 243 K. This indicates that the premelting water forms stronger hydrogen bonds than supercooled bulk water.

While SFG spectra probe the vibrational states of the outermost water molecules adjacent to interfaces, X‐ray reflectivity (XRR) is sensitive to density and thickness. From XRR experiments on ice/SiO_2_ interfaces, Engemann et al.^[10]^ deduced the presence of a premelting layer a few nanometers thick with a density of 1.2 g cm^−3^. This density is significantly different from that of liquid bulk water. However, in addition to ordinary liquid water a variety of liquid water and amorphous ice structures was discovered at low temperatures and/or high pressures:[^10^, ^51^, ^52^] low‐density liquid water (LDL, *ρ*=0.92 g cm^−3^), high‐density liquid water (HDL, *ρ*=1.15 g cm^−3^), low‐density amorphous ice (LDA, *ρ*=0.94 g cm^−3^), and high‐density amorphous ice (HDA, *ρ*=1.17 g cm^−3^). Therefore, Engemann et al. proposed a structural relationship between the premelting layer and high‐density liquid or amorphous water structures.

We now compare our results in Figure 3 with the temperature dependence of *D*_t_ for normal and supercooled (238–273 K) liquid bulk water, amorphous solid water (ASW:LDA, 150–160 K), crystalline ice,^[53]^ and HDA.^[54]^ For all clay composites, *D*_t_ is much lower than for supercooled water. On the other hand, values for *D*_t_ are four or five orders of magnitude higher than that for ASW/ice (Figure 3 in Ref. [53]) and HDA.^[54]^ Therefore, we conclude that the interfacial premelting layer is liquid rather than an amorphous solid. This is consistent with the inelastic neutron scattering results by Zanotti et al.^[55]^ In this work, interfacial liquid water was also found when heating Vycor samples with adsorbed water monolayers from 77 to 280 K.^[55]^ At 240 K, they observed that the interfacial liquid water changes from a low‐density to a high‐density liquid. All *D*_t_ values shown in Figure 3 are obtained at temperatures higher than 240 K, that is, *T* ^−1^<4.17×10^−3^ K^−1^. According to Zanotti et al.,^[55]^ this would mean that the premelting water is HDL with almost constant *D*_t_ values, which is contrary to our result. However, supercooled liquid bulk water is composed of spatially and temporally fluctuating LDL and HDL mixtures.[^56^, ^57^] Moreover, in deeply supercooled water droplets, the Nilsson group recently observed a Widom transition by using femtosecond X‐ray laser pulses.^[52]^ Even at ambient conditions above the Widom line, LDL fluctuations exist in primarily HDL water. And this LDL component can adsorb to solid interfaces. Additionally, neutron diffraction and MD simulations by Soper and Ricci^[58]^ and Mishima and Stanley^[59]^ have shown that LDL is more “structured” and HDL is more “liquid‐like”. This implies a slower diffusion for LDL compared to HDL. Therefore, we suggest that the premelting layer close to solid surfaces contains more “structured” LDL. This interpretation is also consistent with the results obtained from SFG by Sánchez et al.^[12]^

**Figure 4:**
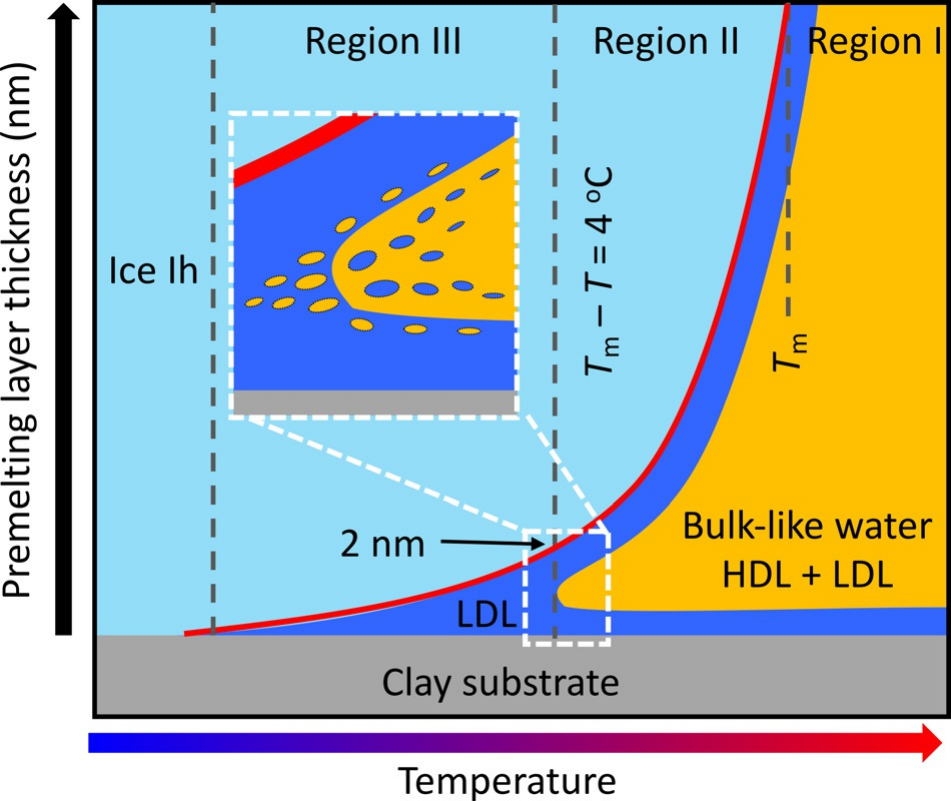
Schematic picture of the structural evolution of ice confined in clay mineral materials when warming it up. The red line shows the growth law of the premelting layer thickness for kaolin, adapted from a high‐energy X‐ray diffraction study using the same kaolin batch.^[41]^ The light‐blue area represents ice Ih, the dark‐blue area LDL, and the orange area bulk‐like liquid water composed of an LDL+HDL mixture. Spatial and temporal fluctuations into LDL and HDL phases appear on either side of the transition (inset).

Based on the above discussion, we propose a picture for the structural evolution of ice confined in porous clay minerals (Figure 4). Upon heating, ice Ih confined in clay minerals forms a premelting liquid. Up to *T*_m_−*T*=4 K, the thickness of the interfacial liquid layer is around 2 nm or less. This liquid consists primarily of the more “structured” LDL water since HDL fluctuations are suppressed adjacent to ice and clays. At higher temperatures, an HDL fraction with faster translational diffusion starts to emerge in regions where water molecules are further apart from the clay and ice surfaces. Spatial and temporal fluctuations between the LDL and HDL components lead to a continuous increase of the diffusion constant as shown in Figure 3 rather than a sharp transition.

This interfacial premelting mechanism has the following implications: The very low friction observed at ice/solid interfaces is explained by the presence of a thin film of liquid water. Aside from ice melting caused by energy dissipation from friction, this liquid film is also caused by the intrinsic interfacial premelting layer studied in this work. Frictional forces are controlled by surface roughness and the viscosity of the interfacial water *η*_i_=*k*_B_ *T*/(6π*D*_t_ *r*). The latter is directly linked to the diffusion by the Stokes–Einstein relation. Experiments determining the interfacial water viscosity from frictional forces yield values for *η*_i_ up to a factor of 20 higher than that of supercooled bulk water *η*_b_.^[22]^ In contrast, our QENS study gives values *η*_i_/*η*_b_≤2. This quotient is about one order of magnitude smaller than calculated from shear forces in friction experiments assuming ideally smooth interfaces. This indicates the importance of surface roughness for a quantitative description of friction at ice/solid interfaces. Therefore, our results contribute to the understanding of friction at ice/solid interfaces.

Furthermore, the viscosity within the interfacial premelting layer can affect the mechanical properties of permafrost. In such partially frozen ice/mineral composites, materials properties are not only determined by the sum of the individual components. For composite engineering materials, it is known that interfaces connecting filler and matrix can be significant. Likewise, the viscosity and thickness of the interfacial premelting layer affects the adhesion between mineral particles and ice. Therefore, the premelting layer is expected to affect the viscoelastic properties of permafrost. Moreover, interfaces can be important for impurity migration in composite materials. For instance, the mobility of plant nutrients and contaminants inside minerals and ice crystals is very low. Therefore, their diffusion along interfaces can be the dominating transport mechanism, despite the small volume fraction of the interfacial premelting layer. As the temperature approaches the melting point, the interfacial layer is thickening. In the context of global warming, the viscosity of the premelting layer could therefore gain increasing relevance.

## Conclusion

The temperature dependence of the translational self‐diffusion coefficient *D*_t_ of water within the premelting liquid layer of three different clay/ice nanocomposites was studied by QENS. Three distinct temperature regions were observed. At low temperature *T*_m_−*T*>4 K (region III), a reduced water mobility within the premelting layer compared to supercooled bulk water was obtained. It is suggested that the water molecules within this premelting layer with less than 2 nm thickness form an LDL structure. In this region, *D*_t_ exhibits a clear trend with the water–substrate interaction strength. The mobility slowdown is most pronounced for the charged hydrophilic vermiculite, followed by the kaolin and more hydrophobic talc samples. At *T*_m_−*T*<4 K (region II), in all clays the *D*_t_ value of the premelting liquid strongly increases with temperature. This effect is explained by the decreasing contribution of water molecules located in direct vicinity of the solid/liquid interface as the fraction of HDL is increasing in the premelting layer.

These results will help to understand friction at ice/solid interfaces. Furthermore, relevance is seen for phenomena related to the water mobility in partially frozen soils. Examples include the transport of guest molecules such as plant nutrients or contaminants within the premelting layer and geochemical reactions such as ion exchange processes at ice/mineral interfaces.

## Conflict of interest

The authors declare no conflict of interest.

## Supporting information

As a service to our authors and readers, this journal provides supporting information supplied by the authors. Such materials are peer reviewed and may be re‐organized for online delivery, but are not copy‐edited or typeset. Technical support issues arising from supporting information (other than missing files) should be addressed to the authors.

Supplementary
