# Supplementary material for: Water Mobility in the Interfacial Liquid Layer of Ice/Clay Nanocomposites
Source: Angew Chem Int Ed Engl. 2021 Feb 25;60(14):7697–702. doi: 10.1002/anie.202013125 (PMC8048683; doi:10.1002/anie.202013125)
Supplement: Supplementary file 1 — Supplementary [file ANIE-60-7697-s001.pdf]

Angewandte Chemie
Eine Zeitschrift der Gesellschaft Deutscher Chemiker

Supporting Information

Water Mobility in the Interfacial Liquid Layer of Ice/Clay Nanocomposites

*Hailong Li, Julian Mars, Wiebke Lohstroh, Michael Marek Koza, Hans-Jürgen Butt, and Markus Mezger**

anie_202013125_sm_miscellaneous_information.pdf

Supporting Information

1. Materials and Sample Preparation

Vermiculite nano-platelets were prepared from thermally exfoliated natural vermiculite (Sigma Aldrich) by a physical process. Gross impurities were removed by iteratively dispersing the raw material in deionized water and removing dense minerals by sedimentation. The cleaned vermiculite was grinded to a fine powder consisting of thin platelets. Kaolin (Acros Organics) and talc suspensions (Carl Roth GmbH + Co. KG) were sonicated 5 times in 60 ºC deionized water for 30 minutes. To remove volatile and organic impurities, the dried powders were annealed under N_2 atmosphere for 5 h at 300 ºC (vermiculite), 12 h at 400 ºC (talc) and 3 h at 100 ºC (kaolin), respectively.

2. Specific Surface Area

The specific surface area s of vermiculite, kaolin, and kaolin samples were determined from nitrogen adsorption isotherms (Quantachrome, Autosorb 1) at 77.3 K (Figure S1). Before the measurements, the samples were degassed for 24 h at 130 ºC in high vacuum. The isotherms are dominated by the presence of slit mesopores, a very small micropore fraction, but no signs for macropores. Quantitative analysis of the experimental data in the pressure range $0 < P/P_0 < 0.25$ using a BET slit model gave a specific surface area of 10.5 m^2/g, 10.2 m^2/g and 4.9 m^2/g for vermiculite, kaolin and talc, respectively.

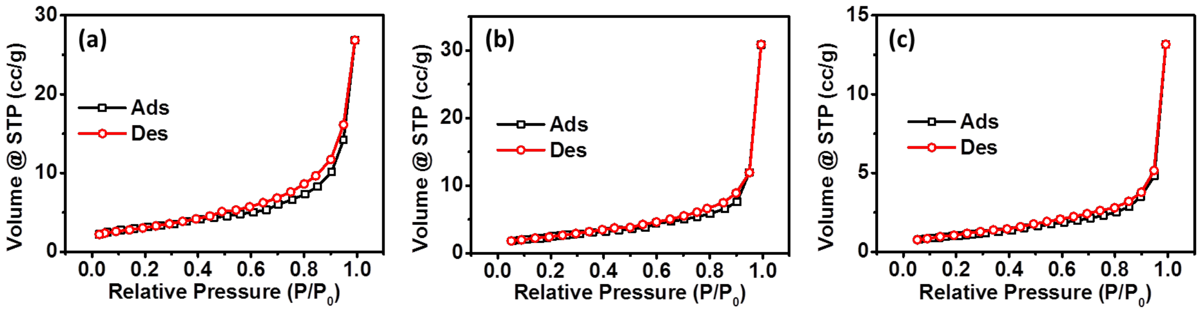

Figure S1. Nitrogen adsorption (black squares) and desorption isotherms (red circles) showing the gas volume V *vs.* relative pressure P/P_0 for (a) vermiculite, (b) kaolin and talc (c) samples.

3. QENS Experiments

Wet clay samples of approx. 0.5 mm thickness were contained in flat, rectangular aluminum cells. Their water content was determined by thermogravimetric analysis (TGA/DSC 3+, Mettler Toledo) after the QENS measurements. We find 33.6 wt.% (vermiculite), 17.6 wt.% (kaolin), 17.4 wt.% (talc I), and 16.2 wt.% (talc II) respectively. This corresponds to an average water thickness of roughly 97 nm (vermiculite), 42 nm (kaolin), 86 nm (talc I), and 79 nm (talc II) between the clay platelets. For these parameters, neutron transmissions of the samples are above or close to 90%. To minimize background scattering, the cell was covered by a cadmium frame leaving a free area of $30 \times 50\,\mathrm{mm}^2$. Temperature was controlled by a cryostat with +/− 0.1 K accuracy.

QENS experiments were performed on the multichopper time-of-flight spectrometer TOFTOF at Heinz Maier-Leibnitz Zentrum (MLZ), Garching, Germany. For all measurements, an incident neutron wavelength λ_0 = 6.0 Å, 14000 rpm chopper speed, and a chopper ratio 5 was used. Measurement times were 5 h to 6 h per temperature. To minimize shadowing effects, the $20 \times 40\,\mathrm{mm}^2$ incident neutron beam impinged the sample under 45°. Resolution functions and flat field references were determined by a 1 mm thick vanadium foil.

The neutron scattering data were processed using the LAMP software.[1,2] The measured neutron intensities were corrected for the energy-dependent neutron detector efficiency, normalized to the vanadium reference, and transformed into scattering vector space and energy transfer $I(q, \omega)$. For better statistics, multiple TOFTOF spectra were grouped.

The experimentally obtained QENS spectra of talc/water and vermiculite/water samples at different temperatures and momentum transfers are presented in Figure S2 and Figure S3.

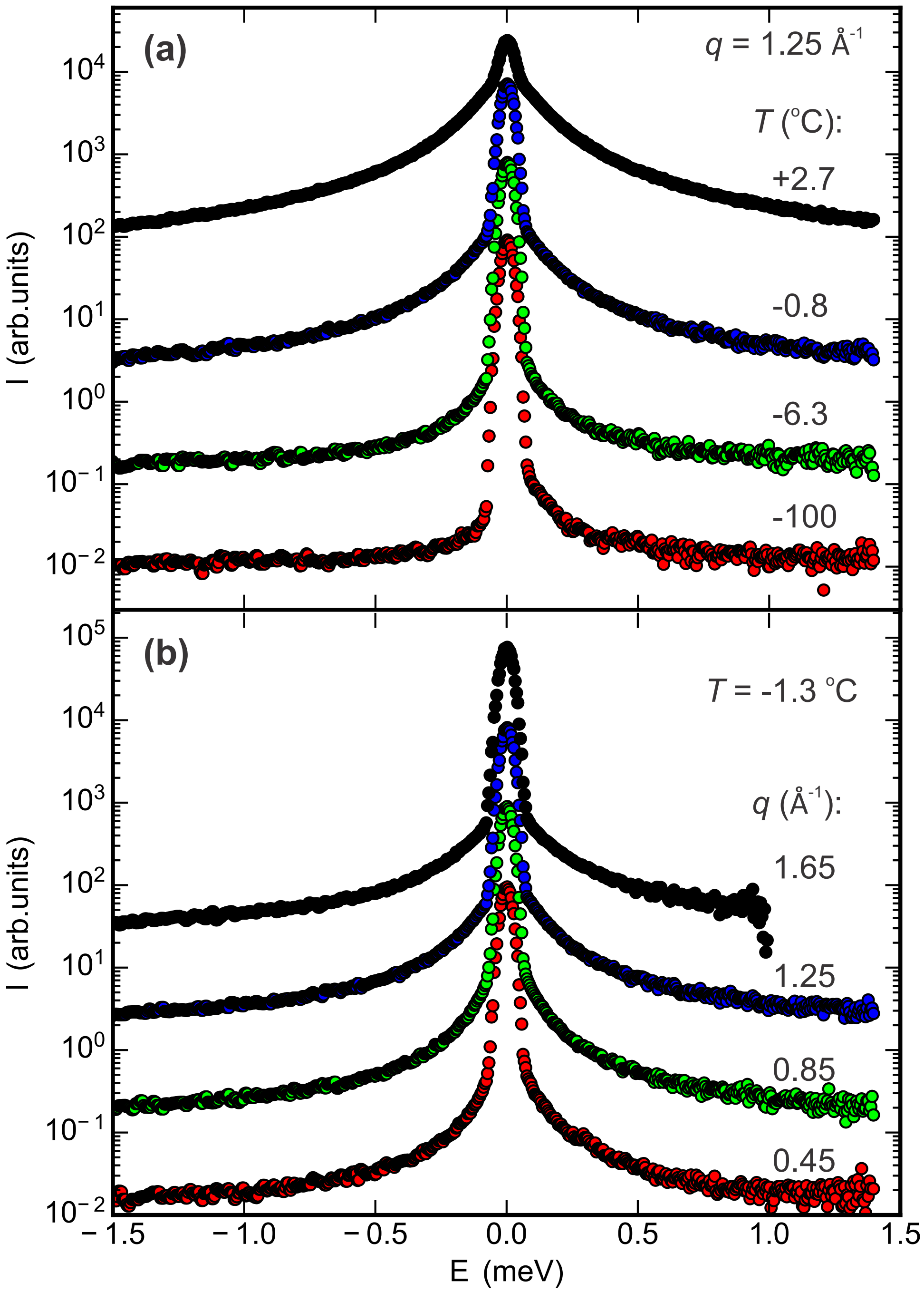

Figure S2. QENS spectra of the talc/water composite sample at momentum transfers q = 1.25 Å^{-1} for temperatures above and below the bulk melting point (a) and at −1.3 °C for different q (b). All curves are vertically shifted for clarity.

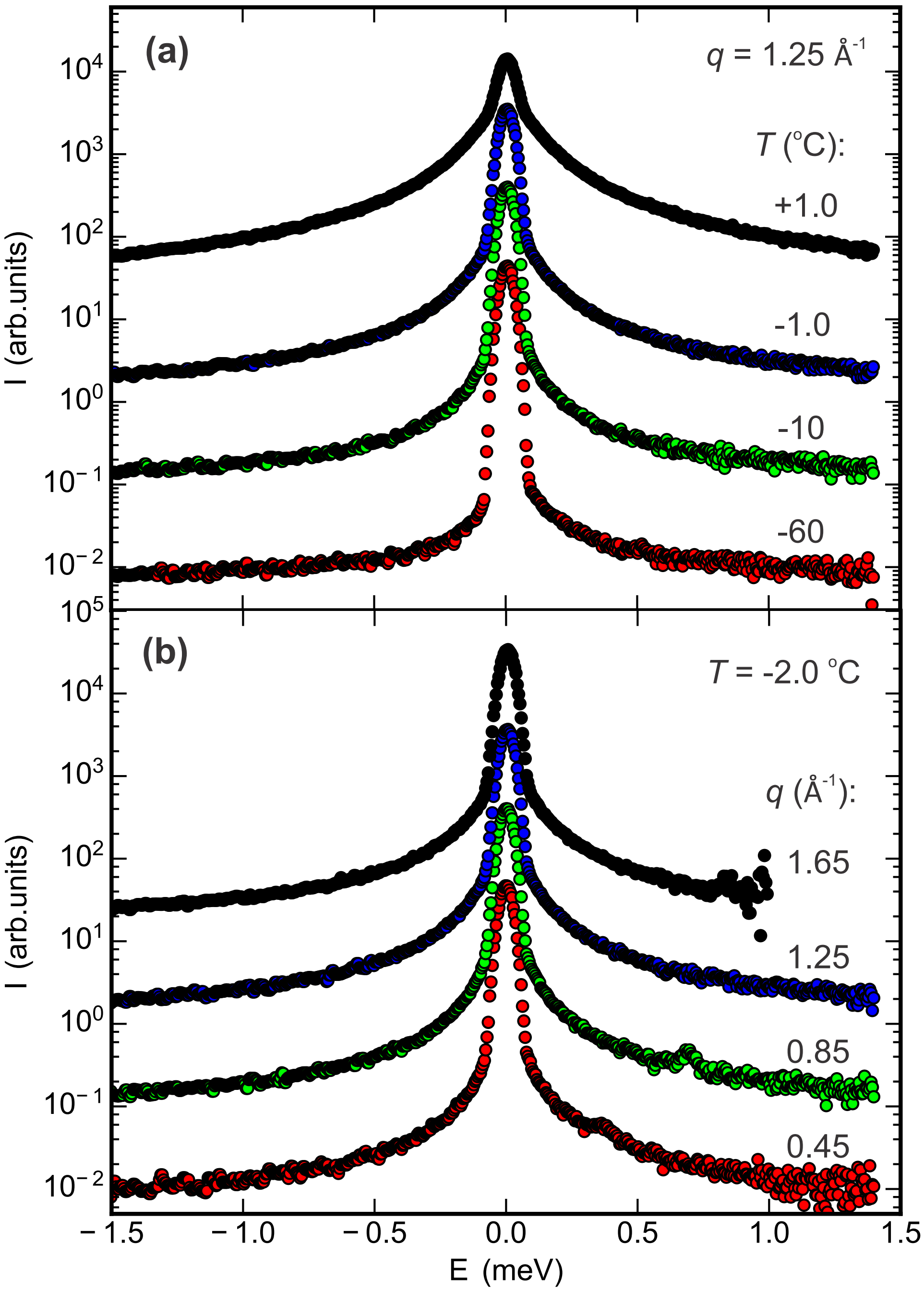

Figure S3. QENS spectra of the vermiculite/water composite sample at momentum transfer $q = 1.25$ Å^{-1} for temperatures above and below the bulk melting point (a) and at −2.0 ºC for different q (b). All curves are vertically shifted for clarity.

4. QENS Analysis

The experimentally obtained QENS intensity $I(q, \omega)$ is given by a convolution of the incoherent dynamic structure factor (DSF) $S(q, \omega)$ with the resolution function $R(q, \omega, \lambda_0)$ multiplied by a scaling factor $a(q, \omega)$.[3,4]

$$I(q, \omega) = a(q, \omega)\, S(q, \omega) \otimes R(q, \omega, \lambda_0) \tag{S1}$$

The resolution of the TOFTOF spectrometer $R(q, \omega, \lambda_0)$ is approximated by a Gaussian function.

$$R(q, \omega, \lambda_0) = \frac{1}{\sigma(q, \lambda_0)\sqrt{2\pi}} \exp\left(-\frac{1}{2}\left[\frac{\omega}{\sigma(q, \lambda_0)}\right]^2\right) \tag{S2}$$

The elastic energy resolution $\boldsymbol{R}(q, \omega)$ is determined by the full width at half maximum (FWHM) of $R(q, \omega, \lambda_0)$:

$$\boldsymbol{R}(q, \lambda_0) = \sqrt{8 \ln 2}\, \sigma(q, \lambda_0) \tag{S3}$$

Values for $\boldsymbol{R}(q, \lambda_0)$ were obtained by fits to vanadium reference spectra recorded at −3.0 °C. Figure S4 summarizes the q-dependent energy resolution at λ_0 = 6 Å. Values are around 50 µeV with a slight increase observed in the q range from 0.3 Å^{-1} to 1.8 Å^{-1}. For comparison, the FWHM of the elastic peak extracted from the kaolin/water sample at −100.0 °C was added to Figure S4. Values extracted from the vanadium reference and the clay show very good agreement.

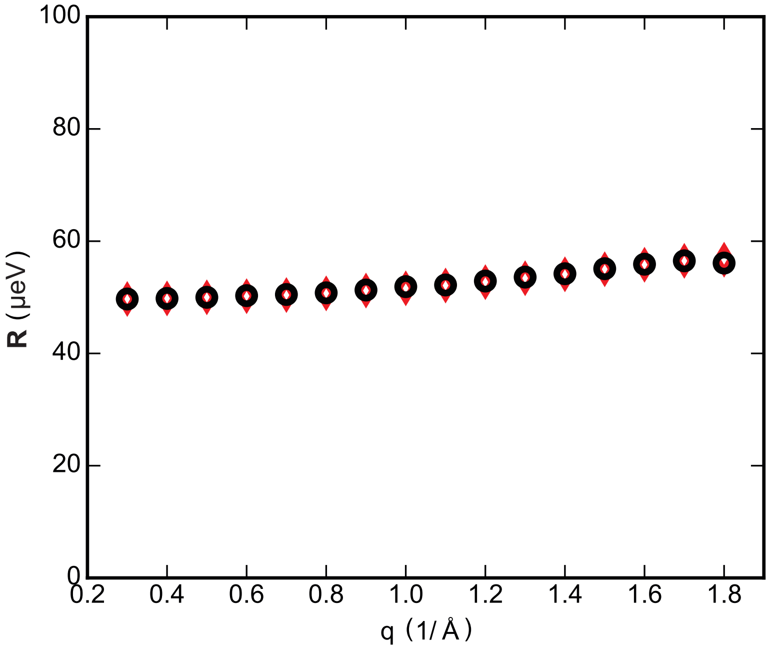

Figure S4. q-dependent energy resolution (FWHM) at λ_0 = 6 Å determined from Gaussian fits to a vanadium reference (black circles) at −3.0 °C and FWHM of the elastic peak of the kaolin/water sample at −100.0 °C (red diamonds).

The scaling factor $a(q,\omega)$ includes the Debye-Waller factor $D_{\mathrm{W}}(q,u)$ and the detailed balance $D_{\mathrm{bal}}(\omega,T)$.

$$a(q,\omega) = D_{\mathrm{W}}(q,u)\, D_{\mathrm{bal}}(\omega,T) = D_{\mathrm{W}}(0)\exp\left(-\frac{1}{3}\langle u^2\rangle q^2\right)\exp\left(\frac{-\hbar\omega}{2k_{\mathrm{B}}T}\right) \qquad \text{(S4)}$$

Here, $\langle u^2\rangle$ accounts for the effective mean square displacement of the protons. Qvist et al. reported values between $\langle u^2\rangle = 0.56^2$ Å^2 and 0.60^2 Å^2.[5] For our QENS analysis a constant average value $\langle u^2\rangle = 0.57^2$ Å^2 was used.

For a quantitative analysis, the QENS spectra were fitted to $S(q,\omega)$ using the approach introduced in the paper of Qvist et al..[5] Here, $S(q,\omega)$ is modeled by a delta function $\delta(\omega)$ for the elastic component of the DSF, two Lorentzians

$$L_{\mathrm{t}}(q,\omega) = \frac{1}{\pi}\frac{\Gamma_{\mathrm{t}}(q)}{\Gamma_{\mathrm{t}}(q)^2+\omega^2} \qquad \text{(S5)}$$

$$L_{\mathrm{r}}(q,\omega) = \frac{1}{\pi}\frac{\Gamma_{\mathrm{r}}(q)}{\Gamma_{\mathrm{r}}(q)^2+\omega^2} \qquad \text{(S6)}$$

plus an energy independent constant term $C(q)$. Here, $L_{\mathrm{t}}(q,\omega)$ and $L_{\mathrm{r}}(q,\omega)$ represent the slow translational and fast rotational diffusions of water molecules, respectively. These two processes contribute with a narrow and broad Lorentzian to the quasi elastic peak. Thus, we get

$$S(q,\omega) = E_{\mathrm{el}}(q)\delta(\omega) + A(q)L_{\mathrm{t}}(q,\omega) + \big(1-A(q)\big)L_{\mathrm{r}}(q,\omega) + C(q) \qquad \text{(S7)}$$

where $E_{\mathrm{el}}(q)$ is the elastic intensity and $A(q)$ is the elastic incoherent structure factor (EISF). Inspired by the work from Qvist et al.,[5] a linear relationship $A(q) = 1 - A_q q$ with a single fitting parameter A_q was assumed (Figure S5).

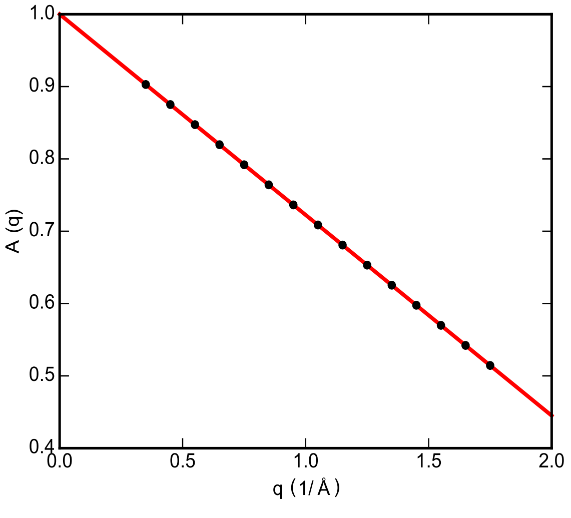

Figure S5. q-dependent EISF (black dots), obtained from fitting spectra of kaolin/water sample at −8.3 °C. The red solid line shows the relations $A(q) = 1 - A_q q$ with $A_q = 0.278$.

Convolution of the Gaussian resolution function with the Lorentz line shape associated with the diffusive processes results in Voigt functions

$$V_\mathrm{n}(q,\omega) \equiv L_\mathrm{n}(q,\omega) \otimes R(q,\omega,\lambda_0) = \frac{\mathrm{Re}[W(Z_\mathrm{n})]}{\sigma(q,\lambda_0)\sqrt{2\pi}} = \frac{\mathrm{Re}\left[\exp\left(-{Z_\mathrm{n}}^2\right)\mathrm{erfc}(-iZ_\mathrm{n})\right]}{\sigma(q,\lambda_0)\sqrt{2\pi}} \quad \text{(S8)}$$

where $Z_n = (\omega + i\Gamma_n)/(\sqrt{2}\sigma)$. $\mathrm{Re}[W(Z_\mathrm{n})]$ is the real part of the Faddeeva function evaluated for Z_n. Here, n indicates the Lorentzian components with width Γ_t and Γ_r.

Substituting Eq. S2, Eq. S4, Eq. S5, Eq. S6, Eq. S7, and Eq. S8 into Eq. S1, gives

$$\begin{aligned} I_\mathrm{total}(q,\omega) &= I_\mathrm{el}(q,\omega) + I_\mathrm{qe}(q,\omega) + B(q) \\ &= D_\mathrm{W}(q,u)E_\mathrm{el}(q)R(q,\omega) + D_\mathrm{W}(q,u)D_\mathrm{bal}(\omega,T)E_\mathrm{qe}(q) \\ &\quad \times \left\{A(q)V_\mathrm{t}(q,\omega) + \left(1 - A(q)\right)V_\mathrm{r}(q,\omega)\right\} + B(q) \end{aligned} \quad \text{(S9)}$$

where $E_\mathrm{qe}(q)$ is the quasi elastic intensity and $B(q)$ is the constant background.

The q-dependent linewidth $\Gamma_\mathrm{t}(q)$ can be described either by the standard diffusion model[6]

$$\Gamma_\mathrm{t}(q) = D_\mathrm{t}q^2 \quad \text{(S10)}$$

where $\mathrm{D_t}$ is the translational diffusion coefficient or by the jump model[3,4]

$$\Gamma_\mathrm{t}(q) = \frac{D_\mathrm{t}q^2}{1 + (ql)^2/6} \quad \text{(S11)}$$

For the QENS analysis, the jump model (Eq. S11) with apparent jump length l = 0.77 Å was chosen.

In particular for low temperatures, the QENS signals from the interfacial premelting layer is comparatively weak. To emphasis these quasi elastic contributions, difference spectra were calculated by subtracting the signals recorded on the completely frozen samples at −100 °C. This procedure efficiently minimizes various background contributions stemming from the clay minerals contained inside a tilted flat-cell sample holder. This includes higher order geometry effects related to the resolution function and the sample transmission that cannot be fully eliminated by standard analysis procedures. For each measured sample and temperature, the parameters for $E_\mathrm{el}(q)$, A_q, $B(q)$, D_t, and Γ_r were determined simultaneously by a consistent fit to all 15 spectra of a data series with q-range from 0.30 Å^{-1} to 1.80 Å^{-1}. Therefore, the values of the

parameter D_t associated with Γ_t was found to be very robust under small variation of the fitting procedure such as the used energy range. To account for the large dynamic range covered by the QENS data, the cost function of the fit was calculated by combined linear and logarithmic scaling of the experimental data. Experimental data for the ice/talc composites was acquired on two individually prepared and characterized samples during two different beamtimes separated by 16 months. In Fig. 3, the parameters extracted from these different experiments are distinguished by green diamonds and green circles. Based on the fluctuations of the results from these two completely independent recorded and analyzed datasets we estimate an overall error of 5% for the diffusion constants.

The q-dependent widths of the peaks contributing to Eq. S9 are plotted in Figure S6 for the kaolin/water sample at −8.3 ºC. The black filled circles show the parameters for the narrow Lorentzian component $\Gamma_t(q)$ used for fitting. For comparison, the blue and red curves show the width $\Gamma_t(q)$ calculated by Eq. S10 and Eq. S11 for the standard and jump diffusion models, respectively. At small q, both models give a similar peak width, smaller than the FWHM of the elastic peak (energy resolution, yellow line). In contrast, for larger $q^2 \geq 1/\text{Å}^2$ significant deviations are found, exceeding 20 % above 1.5 $1/\text{Å}^2$. However, using the jump diffusion model, the experimental data presented in this work is reproduced perfectly over the entire q-range accessible.

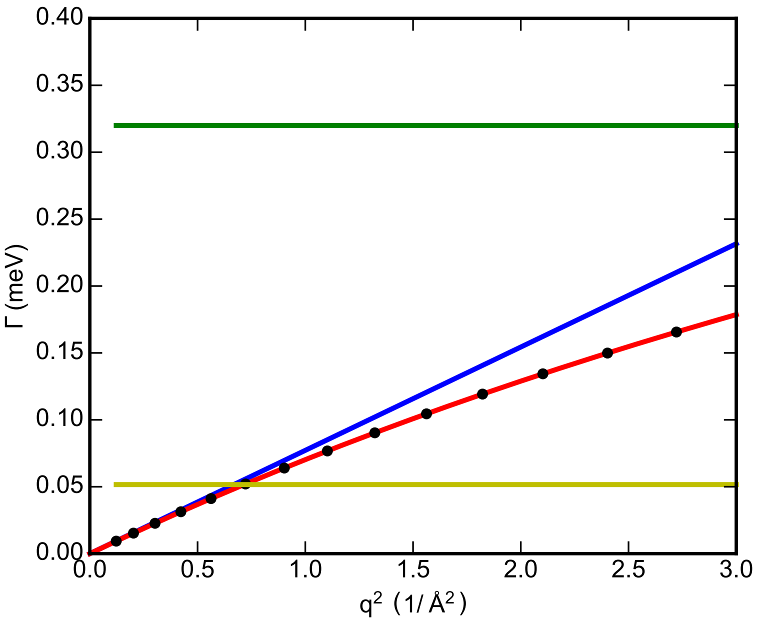

Figure S6. q-dependent width of the narrow Lorentzian component $\Gamma_t(q)$ (black dots), obtained from fitting spectra of kaolin/water sample at −8.3 ºC. The blue and red curves show $\Gamma_t(q)$ calculated using the standard and jump diffusion models. For comparison, the width of the broad Lorentzian component Γ_r (green line) and the energy resolution (yellow line) was added.

References

[1] LAMP, the Large Array Manipulation Program. http: //www.ill.eu/data_treat/lamp/the-lamp-book/**.**

[2] D. Richard, M. Ferrand, G. J. Kearley, *J. Neutron Res.* **1996**, *4*, 33–39.

[3] S. W. Lovesey, Theory of Neutron Scattering from Condensed Matter, Clarendon, Oxford, **1984**, Vol. 1.

[4] M. Bée, Quasielastic Neutron Scattering, Hilger, Bristol, **1988**.

[5] J. Qvist, H. Schober, B. Halle, *J. Chem. Phys.* **2011**, *134*, 144508.

[6] J. Teixeira, M.-C. Bellissent-Funel, S.-H. Chen, A.-J. Dianoux, *Phys. Rev. A* **1985**, *31*, 1913.
